# Ping‐Pong Energy Transfer in Covalently Linked Porphyrin‐MoS_2_ Architectures

**DOI:** 10.1002/anie.201914494

**Published:** 2020-01-30

**Authors:** Ruben Canton‐Vitoria, Tobias Scharl, Anastasios Stergiou, Alejandro Cadranel, Raul Arenal, Dirk M. Guldi, Nikos Tagmatarchis

**Affiliations:** ^1^ Theoretical and Physical Chemistry Institute National Hellenic Research Foundation 48 Vassileos Constantinou Avenue 11635 Athens Greece; ^2^ Department of Chemistry and Pharmacy & interdisciplinary Center for Molecular Materials (ICMM) Friedrich-Alexander Universität Erlangen-Nürnberg Egerlandstrasse 3 91058 Erlangen Germany; ^3^ Universidad de Buenos Aires Facultad de Ciencias Exactas y Naturales Departamento de Química Inorgánica Analítica y Química Física Pabellón 2, Ciudad Universitaria C1428EHA Buenos Aires Argentina; ^4^ CONICET—Universidad de Buenos Aires Instituto de Química-Física de Materiales Medio Ambiente y Energía (INQUIMAE) Pabellón 2, Ciudad Universitaria C1428EHA Buenos Aires Argentina; ^5^ Laboratorio de Microscopias Avanzadas (LMA) Instituto de Nanociencia de Aragon (INA) U. Zaragoza Mariano Esquillor s/n 50018 Zaragoza Spain; ^6^ Instituto de Ciencias de Materiales de Aragon CSIC-U. de Zaragoza Calle Pedro Cerbuna 12 50009 Zaragoza Spain; ^7^ ARAID Foundation 50018 Zaragoza Spain

**Keywords:** 1,2-dithiolane, hybrid materials, MoS_2_, porphyrins, sensitizers

## Abstract

Molybdenum disulfide nanosheets covalently modified with porphyrin were prepared and fully characterized. Neither the porphyrin absorption nor its fluorescence was notably affected by covalent linkage to MoS_2_. The use of transient absorption spectroscopy showed that a complex ping‐pong energy‐transfer mechanism, namely from the porphyrin to MoS_2_ and back to the porphyrin, operated. This study reveals the potential of transition‐metal dichalcogenides in photosensitization processes.

## Graphical abstract

**Two‐way street**: A hybrid porphyrin‐MoS_2_ material has been shown to undergo complex ping‐pong energy transfer from the porphyrin to the MoS_2_ and back to the porphyrin. This finding illustrates the potential of transition‐metal dichalcogenides in photosensitization processes.

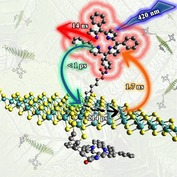

Molybdenum disulfide (MoS_2_) is a typical example of a layered transition‐metal dichalcogenide. In general, the structure of such dichalcogenides is analogous to that of graphene, with an atomic layer of a transition metal sandwiched between two layers of chalcogen atoms. More specifically, each Mo atom is bound to six S atoms and forms a three‐atom‐thick monolayer.^1^ Exfoliated MoS_2_ materials have received enormous attention in recent years because of their extraordinary optoelectronic and electrocatalytic properties, especially in the area of energy applications and catalysis.^2^, ^3^ Diverse exfoliation strategies have been developed for MoS_2_. Most notable is the employment of a) strong intercalants such as Li^+^, which result in phase transfer to an octahedral metallic 1T polytype,^4^ and b) non‐oxidative Brønsted acids such as chlorosulfonic acid, which offer the benefit of retaining the 2H semiconducting phase.^5^

Additional efforts have been placed on developing strategies for chemically modifying transition‐metal dichalcogenides.^6^ The aim has been to fully harness the properties of, for example, MoS_2_. In fact, incorporation of molecular dopants results in fine‐tuning of the electronic as well as optical properties of MoS_2_ which, in turn, enables broadening the spectrum of its applications. In MoS_2_, the S atoms in the basal plane are, however, rather inert as a result of saturation. Furthermore, Mo atoms are placed between S layers and, thereby have challenging chemical reactivity. However, the covalent functionalization of MoS_2_ has been accomplished.^7^ For example, the basal plane of the 1T‐MoS_2_ polytype has been reacted with organoiodides,^8^ while reaction with diazonium salts has been reported for the basal plane modification of 1T^9^ as well as 2H‐MoS_2_.^10^ Herein we adopt the recently developed method based on the addition of 1,2‐dithiolanes to S‐vacant sites located at the edges of exfoliated 2H‐MoS_2_ nanosheets.^11^ A true advantage of this approach is that diverse 1,2‐dithiolanes are easily prepared and they react with exfoliated MoS_2_ to yield interesting hybrids. Pyrene and phthalocyanines have, for example, been coupled with MoS_2_.^11^ Alternatively, ammonium moieties have been grafted onto MoS_2_ and WS_2_, which facilitated the electrostatic association of carbon dots^12^ and anionic porphyrins.^13^ In the resulting ensembles, excited‐state electronic interactions between the components were responsible for quenching the fluorescence from the pyrene, carbon dots, or porphyrin. The major drawbacks of electrostatically associated ensembles are their lack of stability and processability in organic solvents. They also have moderate binding strengths, especially when compared to the robust and strong bonding in covalently linked conjugates. Therefore, it is not only imperative, but also timely, to explore covalent modification of MoS_2_ with photoactive porphyrins.

Herein, we report on the modification of the edges of exfoliated MoS_2_ with a 1,2‐dithiolane derivative **1** featuring a porphyrin (H_2_P). The newly prepared H_2_P‐MoS_2_ **2** was comprehensively characterized by spectroscopic, thermal, and microscopy means. We also gathered insight into the electronic interactions between the porphyrin and MoS_2_ on the femto‐ to nanosecond timescales upon photoexcitation.

1,2‐Dithiolane‐based porphyrin **1** was synthesized by a condensation reaction between α‐lipoic acid and 5‐(4‐aminophenyl)‐10,15,20‐(triphenyl)porphyrin. In parallel, treatment of bulk MoS_2_ with chlorosulfonic acid allowed exfoliation of semiconducting nanosheets.^5^ Next, the reaction of exfoliated MoS_2_ with **1** yielded H_2_P‐MoS_2_ **2**, as summarized in Figure 1. Filtration of the reaction mixture through a PTFE membrane (0.2 μm pore size) followed by extensive washing with dichloromethane, assured the removal of any noncovalently physisorbed **1**, as revealed by UV/Vis spectroscopy (Figure S1). Purified **2** showed reasonable solubility in DMF, benzonitrile, and 2‐propanol, while it is completely insoluble in dichloromethane and water.

**Figure 1:**
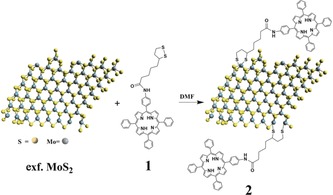
Reaction route for obtaining H_2_P‐MoS_2_ **2**.

Evidence for the success in the covalent modification of MoS_2_ with H_2_P came from vibrational spectroscopy. Attenuated total reflectance infrared (ATR‐IR) measurements on H_2_P‐MoS_2_ **2** (Figure 2 a) revealed the presence of a) C−H stretching vibrations corresponding to the alkyl chain, which connects H_2_P with MoS_2_, at 2848 and 2950 cm^−1^, b) an amide carbonyl vibration at 1660 cm^−1^, and c) an aromatic C=C bending at 1590 cm^−1^. We further validated the origin of the IR bands related to H_2_P within the hybrid by performing a reference experiment in which a mixture of tetraphenylporphyrin and exfoliated MoS_2_ was processed under the same experimental conditions as those for obtaining **2**. The presence of physisorbed tetraphenylporphyrin was not detected in the UV/Vis and emission spectra of the reference material, and no contribution in the mass loss detected by TGA was observed (Figures S2–S4).

**Figure 2:**
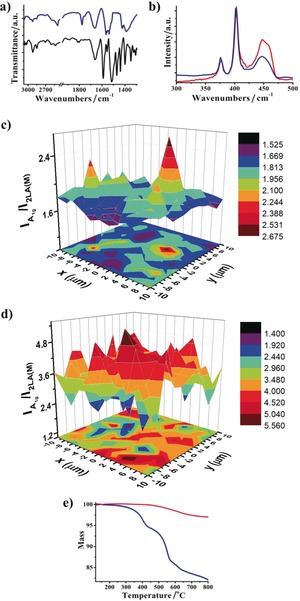
a) ATR‐IR spectra of **1** (black) and **2** (blue). b) Raman spectra of exfoliated MoS_2_ (red) and **2** (blue) upon excitation at *λ*=633 nm. c, d) Raman mapping upon excitation at *λ*=633 nm of the *I* ${}_{A{}_{1g}}$ /*I*_2LA(M)_ intensity ratio of a 20× 20 μm^2^ area for c) exfoliated MoS_2_ and d) **2**. e) TGA plots of exfoliated MoS_2_ (red) and **2** (blue).

Additional structural information regarding **2** came from Raman spectroscopy. Excitation at *λ*=514 nm showed the existence of the C=C and C=N bonds of H_2_P in the 1000–1600 cm^−1^ region (Figure S5). Characteristic modes corresponding to MoS_2_ were observed in the lower wavenumber region, especially upon excitation at *λ*=633 nm. These modes become visible as a result of coupling with the A1 excitonic transition, which in turn produces resonance Raman enhancement of the first and second order vibrational modes. Exfoliated semiconducting MoS_2_ gave rise to bands corresponding to 2LA(M) at 447 cm^−1^, A_1g_ at 406 cm^−1^, and $E_{2g}^{1}$ at 382 cm^−1^ (Figure 2 b). The frequency difference between A_1g_ and $E_{2g}^{1}$ was 24 cm^−1^, from which we conclude the existence of few‐layered MoS_2_.^14^, ^15^ The 2LA(M) vibration relates to the S‐vacancies.^16^ By comparing the Raman spectrum of exfoliated MoS_2_ with that of **2**, the intensity ratio of A_1g_ compared to 2LA(M) was found to be considerably higher for **2**, that is, 3.6 versus 1.9 for exfoliated MoS_2_. Complementary Raman mapping assays revealed an increased *I* ${}_{A{}_{1g}}$ /*I*_2LA(M)_ intensity ratio for **2** compared to that of exfoliated MoS_2_ (Figure 2 c,d). This observation is in sharp contrast to the recorded *I* ${}_{A{}_{1g}}$ /*I*_2LA(M)_ maps for the reference material prepared by physisorption (Figure S6). Overall, this is indicative of a “healing” of the S‐vacancies by the 1,2‐dithiolane species in **2**.^9^ Likewise, the absence of features at 150, 225, and 325 cm^−1^, indicative of the metallic polytype, highlights the semiconducting nature of MoS_2_ in **2**.

Thermogravimetric analysis (TGA) provided information related to the degree of MoS_2_ functionalization in **2**. Exfoliated MoS_2_ was found to be thermally stable under N_2_ from 100 to 800 °C. Notably, the mass loss observed in the modified MoS_2_ is directly related to the thermal decomposition of the organic addends. Hence, the mass loss of 9 % for **2** (Figure 2 e), which occurred up to 520 °C, is ascribed to the porphyrin. Based on this mass loss, a loading of one H_2_P moiety per 54 units of MoS_2_ was calculated. This is in good agreement with values reported for similar functionalizations of MoS_2_ with pyrene and zinc phthalocyanine.^11^

Spatially resolved electron energy loss spectroscopy (SR‐EELS) using a scanning transmission electron microscope (STEM) enables the morphology and chemical composition of complex hybrid nanostructures to be investigated at the local scale.^17^ Thus, we performed SR‐EELS STEM analyses of **2**. Figure 3 shows an EEL spectrum line (SPLI) of a functionalized MoS_2_ flake (see also Figure S7). Figure 3 a corresponds to the high angular annular dark field (HAADF) STEM image of the flake and the SPLI was acquired along the highlighted green line. Typical EEL spectra are displayed in Figure 3 b. Each of them corresponds to the sum of four EEL spectra extracted from the two areas marked in the SPLI (red (i) and green (ii) lines, respectively). S‐L_2,3_, Mo‐M_4,5_, C‐K, and Mo‐M_2,3_ edges are observed in both spectra. The energy‐loss near‐edge structure (ELNES) investigations of the C‐K edge are displayed in the right panel of Figure S7. Two signatures at 291.2 and 293.4 eV can be observed in the σ* region (ca. 290–325 eV) for **2**. Such signals are not discerned in the spectra of other carbon‐containing materials employed as references. Some differences can also be observed in the main contribution to the π* signal at ≈284–290 eV. Here, the C‐K edge is dominated by an aromatic C contribution (ca. 285.5 eV17b, 17c, 18), which is less pronounced in the case of H_2_P. Furthermore, another feature at about 287.2 eV is clearly seen in the spectra of **2**. This signal can be attributed to pyrrolic (C−N) contributions of H_2_P.18c Importantly, the N‐K edge cannot be observed in H_2_P because the Mo‐M_2,3_ edge is in the same energy range. For all these reasons, the features highlighted in the C‐K edge of **2** correspond to a porphyrin contribution. Furthermore, we conclude from the EELS analyses that H_2_P is homogeneously distributed across the surface of the MoS_2_ flakes in **2** (Figure S8).

**Figure 3:**
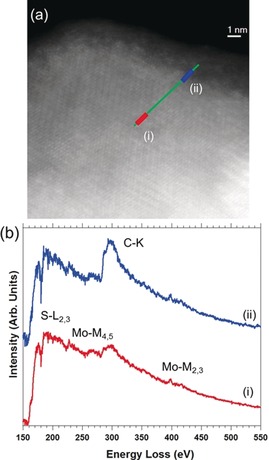
a) HAADF HRSTEM image of a flake of **2**. An EELS SPLI was acquired along the green line. b) Two EEL spectra, each corresponding to the sum of four selected EEL spectra collected from the two marked regions in the SPLI shown in (a). The S‐L_2,3_, Mo‐M_4,5_, C‐K, and Mo‐M_2,3_ edges are visible in both spectra. The carbon detected is associated with H_2_P.

Next, focusing on the optical properties of **2**, the UV/Vis spectrum was recorded in DMF and compared with that of **1**. In particular, the UV/Vis spectrum of **1** showed a sharp absorption at *λ*=418 nm that corresponds to the Soret band, accompanied by weaker Q‐band absorptions at *λ*=516, 550, 596, and 648 nm (Figure 4, left). Spectroscopic fingerprints at *λ*=401, 494 (C‐exciton), 626 (B‐exciton), and 681 nm (A‐exciton) are discernable in the UV/Vis spectrum of exfoliated MoS_2_ (Figure 4, left). The UV/Vis spectrum of **2** is best described as a superimposition of the individual spectra of **1** and exfoliated MoS_2_. The spectrum is, for example, dominated by the characteristic Soret‐band absorption of H_2_P at *λ*=418 nm in addition to the MoS_2_‐related transition at *λ*=679 nm. From this similarity, we deduce that the ground‐state lacks appreciable electronic interactions.

**Figure 4:**
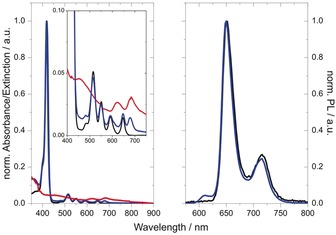
Left: Electronic absorption spectra of **1** (black), exfoliated MoS_2_ (red), and **2** (blue) in DMF. Right: Fluorescence spectra of **1** (black) and **2** (blue) in DMF upon excitation at *λ*=420 nm.

Excited‐state electronic interactions could also be used to probe interactions between the H_2_P and MoS_2_ in **2**. Specifically, strong fluorescence, centered at *λ*=651 and 715 nm (Figure 4, right), was found upon excitation of **1** at the Soret‐band absorption at *λ*=420 nm. A fluorescent lifetime of 10.3 ns (Figure S9) was determined by time‐correlated single photon counting (TCSPC) measurements on **1** with DMF as the solvent. Photoexcitation of **2** at *λ*=420 nm, however, resulted in no significant quenching of the porphyrin‐centered fluorescence relative to that of **1** (Figure 4, right). For **2**, the fluorescence maxima were observed at *λ*=649 and 714 nm, and the fluorescence lifetime was 10.3 ns (Figure S9).

As a complement to the aforementioned method, we probed **1**, exfoliated MoS_2_, and **2** by means of femtosecond transient absorption spectroscopy (fsTAS) using an excitation wavelength of 420 nm (Figure 5, see also Figures S10 and S11). For **1**, bleaching of the ground state at *λ*=517 and 653 nm is observed early in the experiments. In addition, positive absorptions evolved at *λ*=442, 538, 574, 623, and 690 nm (Figure S10). Global analysis revealed the presence of three exponential decays on the fsTAS timescale. These three decays are followed by a much slower decay, whose dynamics are outside the timescale of our experiments. Therefore, a target model was applied to fit the transient absorption data (Figure 6, right). This involves an initial population of a second singlet excited state [S_2_(H_2_P)], consistent with excitation into the Soret‐band absorption, which internally converts into the first singlet excited state [S_1_(H_2_P)] within 16 ps. The latter undergoes intersystem crossing within 10 ns to the triplet state [T_1_(H_2_P)], which, in turn, decays back to the ground state on the microsecond timescale. Additionally, aggregates of **1** are present in the sample and they were found to decay to the ground state within 142 ps. These dynamics resemble very closely those already reported for a variety of porphyrins.^19^, ^20^

**Figure 5:**
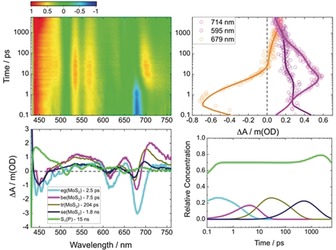
Top left: Differential absorption 3D map of **2** in DMF at room temperature with excitation at *λ*=420 nm. Top right: Time absorption profiles and fits at selected wavelengths. Bottom left: Species‐associated differential spectra of exciton generation (e.g. [MoS_2_]: cyan curve), biexcitons (be[MoS_2_]: pink curve), trions (tr[MoS_2_]: brown curve), single excitons (se[MoS_2_]: dark blue curve), and singlet excited state (S_1_(H_2_P): green curve). Bottom right: Evolution of the different species over time.

**Figure 6:**
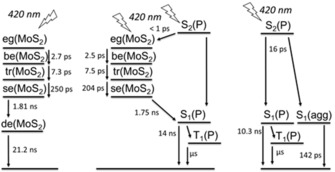
Deactivation models for MoS_2_ (left), **2** (middle), and **1** (right) upon excitation at 420 nm.

For exfoliated MoS_2_, ground‐state bleaching evolved at *λ*=476, 623, and 683 nm together with positive absorptions at *λ*=591, 655, and 743 nm (Figure S11). Global analysis of the data revealed five exponential decays after the absorption of light. The first three decays, which take place within 2.7, 7.3, and 250 ps, involve biexciton [be(MoS_2_)] and trion [tr(MoS_2_)] formation as well as the decay of these many‐body particles into single excitons [se(MoS_2_)]. Single excitons then diffuse within 1.8 ns across the layers in the *z* direction [de(MoS_2_)], before they recombine to recover the ground state within 21.2 ns (Figure 6, left). These dynamics resemble those already reported for exfoliated MoS_2_.^21^

For **2**, excited states of both components are observed to participate in the decay cascade (Figure 5). Selective excitation into the Soret‐band absorption of **2** affords a photoinduced absorption at *λ*=450 nm and ground‐state bleaching of the bands at *λ*=617 and 679 nm. Please note that, while the first absorption belongs to the porphyrin, the last two are fingerprints of MoS_2_. Considering that porphyrin absorptions in **2** are around 20 times stronger than those of MoS_2_ at the excitation wavelength (Figure 4, left), the similar intensities of the differential features at *λ*=450 and 679 nm imply an ultrafast energy transfer (<1 ps) from S_2_, which is populated upon excitation at *λ*=420 nm, to MoS_2_. However, since the energy transfer is faster than the time resolution of our instrument, we are unable to measure the population of the S_2_(H_2_P). Global analysis showed four exponential decays on the fsTAS timescale and an additional decay, which is, however, outside of our detection range. Our interpretation is based on a kinetic target model, which is depicted in the middle panel of Figure 6. This considers an initial excited state population governed by a porphyrin‐centered S_2_, with a minor contribution from a MoS_2_‐centered excited state. A nonquantitative ultrafast energy transfer from H_2_P to MoS_2_ then occurs, which results in comparable populations of the H_2_P‐centered (70 %) and MoS_2_‐centered (30 %) excited states. Afterwards, these three species, which govern the deactivation of exfoliated MoS_2_ and which result in the formation of single excitons, are discernible. The times for the interconversions are 2.5, 7.5, and 204 ps. Once populated, the single exciton of MoS_2_ is subject to a second energy transfer. This time, however, single excitons are transferred from the MoS_2_ to the S_1_ state of H_2_P. This energy transfer takes 1.75 ns. From here, the porphyrin decay proceeds through intersystem crossing to the corresponding triplet excited state within 14 ns, and recovery of the ground state on the microsecond timescale. This mechanism is also based on the energetics of **2**. The S_1_ state of **1**, with its fluorescence at 1.73 eV (*λ*=715 nm) and the MoS_2_ fluorescence at 1.82 eV,^22^ render the second energy transfer a downhill process. This ping‐pong energy‐transfer model enables us to rationalize the nearly identical steady‐state photophysical characteristics of **1** and **2** upon photoexcitation.

In summary, we accomplished the covalent grafting of porphyrin **1** onto exfoliated MoS_2_ to afford novel H_2_P‐MoS_2_ **2**, which was fully characterized. Thorough photophysical investigations based on steady‐state and time‐resolved measurements corroborate that the decay of the photoexcited porphyrin involves a ping‐pong energy transfer to and from MoS_2_. Our findings suggest that transition‐metal dichalcogenides have great potential in photosensitization. Such hybrid materials may be useful in energy‐conversion applications.

## Conflict of interest

The authors declare no conflict of interest.

## Supporting information

As a service to our authors and readers, this journal provides supporting information supplied by the authors. Such materials are peer reviewed and may be re‐organized for online delivery, but are not copy‐edited or typeset. Technical support issues arising from supporting information (other than missing files) should be addressed to the authors.

Supplementary
