# Supplementary material for: Ping‐Pong Energy Transfer in Covalently Linked Porphyrin‐MoS2 Architectures
Source: Angew Chem Int Ed Engl. 2020 Jan 30;59(10):3976–81. doi: 10.1002/anie.201914494 (PMC7154652; doi:10.1002/anie.201914494)
Supplement: Supplementary file 1 — Supplementary [file ANIE-59-3976-s001.pdf]

Angewandte Chemie
Eine Zeitschrift der Gesellschaft Deutscher Chemiker

Supporting Information

Ping-Pong Energy Transfer in Covalently Linked Porphyrin-MoS_2 Architectures

Ruben Canton-Vitoria[+], Tobias Scharl[+], Anastasios Stergiou, Alejandro Cadranel, Raul Arenal, Dirk M. Guldi,* and Nikos Tagmatarchis**

anie_201914494_sm_miscellaneous_information.pdf

Supporting Information

EXPERIMENTAL SECTION

All chemical and solvents were purchased from Sigma Aldrich and used as received. 5-(4-aminophenyl)-10,15,20-triphenyl-21H,23H-porphin was purchased from TriPorTech GmbH. ^{1}H and^{13}C NMR spectra were recorded in a 300 MHz Varian instrument operated by Vjnmr software, with TMS used as internal standard and D_2O or $CDCl_3$ as solvent All chemicals were used as received without further purification. Tip sonication was performed with a Bandelin Sonoplus Ultrasonic Homogenizer HD 3200 equipped with a flat head probe (VS70T), running at 35% of the maximum power (250 W). UV-Vis absorption spectra were recorded on a PerkinElmer (Lambda 19) UV-Vis-NIR spectrophotometer. Steady-state emission spectra were recorded on a Fluorolog-3 JobinYvon-Spex spectrofluorometer (model GL3-21). Pico-second time-resolved fluorescence spectra were measured by the time-correlated-single-photon-counting (TCSPC) method on a Nano-Log spectrofluorometer (Horiba JobinYvon), by using a laser diode as an excitation source (NanoLED, 375 nm) and a UV-Vis detector TBX-PMT series (250-850 nm) by Horiba JobinYvon. Lifetimes were evaluated with the DAS6 Fluorescence-Decay Analysis Software. Mid-infrared spectra in the region 500−4500 cm^{-1} were obtained on a Fourier transform IR spectrometer (Equinox 55 from Bruker Optics) equipped with a single reflection diamond ATR accessory (DuraSamp1IR II by SensIR Technologies). A drop of the solution was placed on the diamond surface, followed by evaporation of the solvent, in a stream of nitrogen, before recording the spectrum. Typically, 100 scans were acquired at 2 cm^{-1} resolution. Micro-Raman scattering measurements were performed at room temperature in the backscattering geometry using a RENISHAW in Raman microscope equipped with a CCD camera and a Leica microscope. For the Raman spectroscopic studies, all samples were deposited in the form of dried powder on glass microscope slides. In all cases, the exposure time was 10s and the employed lens was a

long working distance x50 objective. The power of the 514 and 633 nm laser lines was maintained below 0.5 mW (~0.4 mW for 633 nm and ~0.1 mW for 514 nm). For the 514 nm measurements a 2400 mm^{-1} grating was used, under extended mode. For the 633 nm measurements a 1200 mm^{-1} grating was used (both for static and extended modes). For mapping studies at 633 nm, a 400 μm^2 area was scanned with a step of 2 μm following a raster sampling pattern, under static mode (centered at 420 cm^{-1}). Raman spectra were collected on numerous spots on the sample and recorded with Peltier cooled CCD camera. The data were collected and analyzed with Renishaw Wire and Origin software. Thermogravimetric analysis was performed using a TGA Q500 V20.2 Build 27 instrument by TA in a nitrogen (purity >99.999%) inert atmosphere. Scanning Electron Microscope (SEM) imaging and Energy Dispersive X-ray Spectroscopy (EDS) were performed using a FE-SEM (model JSM-7610F) equipped with an EDAX (X-ACT, Oxford instrument).

For TEM measurements, samples were prepared by dissolving the MoS_2 flakes in ethanol and bath sonicating. A droplet of this solution was deposited on holey carbon TEM cupper grids. Spatially-resolved electron energy loss spectroscopy (SR-EELS) measurements were performed on probe-corrected scanning TEM (STEM) FEI Titan Low-Base operating at 80 kV (fitted with a X-FEG® gun and Cs-probe corrector (CESCOR from CEOS GmbH)). EEL spectra were recorded using the spectrum-imaging (SPIM in 2D or spectrum-line (SPLI) in 1D) mode[1,2] in a Gatan GIF Tridiem ESR 865 spectrometer. The convergent semi-angle was of 25 mrad, the collection semi-angle was 80 mrad and the energy resolution ~ 1.2 eV. The EELS datasets were denoised with the open-source program Hyperspy by using principal component analysis.[2] Furthermore, to avoid the effects of electron beam damage, particular attention was paid to reduce the beam dose used to optimize the acquisition conditions.

The exfoliated MoS_2 and H_2P-MoS_2 samples for optical measurements were suspended in DMF, sonicated for 150 minutes, and afterwards centrifugated for 10 min at 14000 rpm. Steady state absorption and fluorescence spectroscopy were performed with a Lambda 2 from Perkin Elmer and a Fluoromax-3 from Horiba, respectively. Ultrafast transient absorption (TA) experiments were conducted using an amplified Ti:sapphire fs laser system (Clark MXR CPA2101 and 2110, FWHM = 150 fs, λexc = 420 nm, 200 nJ per pulse) with TA pump / probe Helios detection systems from Ultrafast Systems. White light was generated focusing a fraction of the fundamental 775 nm output onto a 2 mm sapphire disk. A magic angle configuration was employed to avoid rotational dynamics. Excitation pulses of 420 nm wavelength were generated by a NOPA with subsequent frequency doubling. Bandpass filters with ± 5 or ± 10 nm were used to ensure low spectral width and to exclude 775 and 387 nm photons. All measurements were conducted in a 2 mm quartz cuvettes under argon atmosphere. Obtained data were treated by global analyses using the R- package TIMP and GloTarAn.

Exfoliated MoS_2. Chlorosulfonic acid (2 mL) was added to bulk MoS_2 (150 mg) and the mixture was sonicated for 2hrs. In the resultant raw dispersion, distilled water was carefully added under vigorous stirring at 0 ^oC, until the production of fuming HCl stopped. In the next step, the dispersion was filtered over PTFE filter (pore size 0.2 μm) and washed several times with distilled water. Before the filter was completely dried, the residual material was transferred to a beaker, NMP (150 mL) was added and the mixture was probe-sonicated (1hr, 35% amplitude, 0 ^oC). The material was left overnight to settle and the exfoliated MoS_2 was obtained from the supernatant. The 2/3 of the gray-greenish supernatant dispersion were carefully collected, filtered over PTFE filter (pore size 0.2 μm) and washed several times with distilled water and finally with methanol and dichloromethane. The exfoliated MoS_2 material was obtained as a dry lead-grey powder.

Synthesis of 1. In a round bottom flask, α- lipoic acid (1.6 mmol), tert-butyl (2-(2-(2-aminoethoxy)ethoxy)ethyl)carbamate (4.8 mmol, 3 equiv.), 4-dimethylaminopyridine (4.8 mmol, 3 equiv.) and 4-(10,15,20-triphenylporphyrin-5-yl)aniline (1.6 mmol) were added in dry dichloromethane (100 mL). The reaction mixture was stirred under nitrogen atmosphere at room temperature for 18 hours. Then, the organic phase was extracted with H_2O (5 x 100 mL), dried over $MgSO_4$ and purified by column chromatography (petroleum ether/ethyl acetate 50%). ^1H NMR (300 MHz, $CDCl_3$) δ 8.84 (m, 8H), 8.20 (m, 8H), 7.93 – 7.81 (m, 2H), 7.77 (s, 9H), 7.48 (s, 1H), 4.27 (s, 1H), 3.53 (ddd, J = 22.3, 12.4, 5.4 Hz, 2H), 2.75 – 2.63 (m, 1H), 2.54 (t, J = 7.2 Hz, 2H), 2.36 (s, 1H), 2.05 – 1.45 (m, 6H). ^{13}C NMR (300 MHz, $CDCl_3$) δ 171.38 (1C), 142.19 (8C) 138.04 (2C) 137.66 (3C) 135.12 (2CH), 134.60 (4CH), 131.21 (8CH), 127.79 (3CH), 126.76 (6CH), 120.26 (2CH), 118.04 4x(CH), 56.49 (CH), 40.34 (CH_2), 38.56 (CH_2), 34.76 (CH_2), 29.77 (CH_2), 28.97 (CH_2), 25.35 (CH_2). ESI-HRMS calcd for $C_{52}H_{43}ON_5S_2$: 817,2909, found: m/z 840.28006 $[M+Na]^+$.

Preparation of hybrid material 2. In a round bottom flask, exfoliated MoS_2 (15 mg) and **1** (10 mg) in DMF (10 mL) were stirred at 75 °C for 72 hours. After that period, the reaction mixture was filtered through a PTFE membrane (0.2 μm pore size), the solid residue was washed with DMF and dichloromethane to obtain material **2a** as lead-grey powder. In order to detect any traces left of possibly physisorbed compound **1**, the isolated solid was redispersed in DMF with the aid of mild sonication, followed by centrifugation. The absence of absorption features owed to free porphyrin in the UV-Vis spectra of the supernatant implied the successful purification of the hybrid material.

References

[1] C. Jeanguillaume, C. Colliex, *Ultramicroscopy* **1989**, *28*, 252-257.

[2] R. Arenal, F. de la Peña, O. Stephan, M. Walls, M. Tence, A. Loiseau, C. Colliex, *Ultramicroscopy* **2008**, *109*, 32-38.

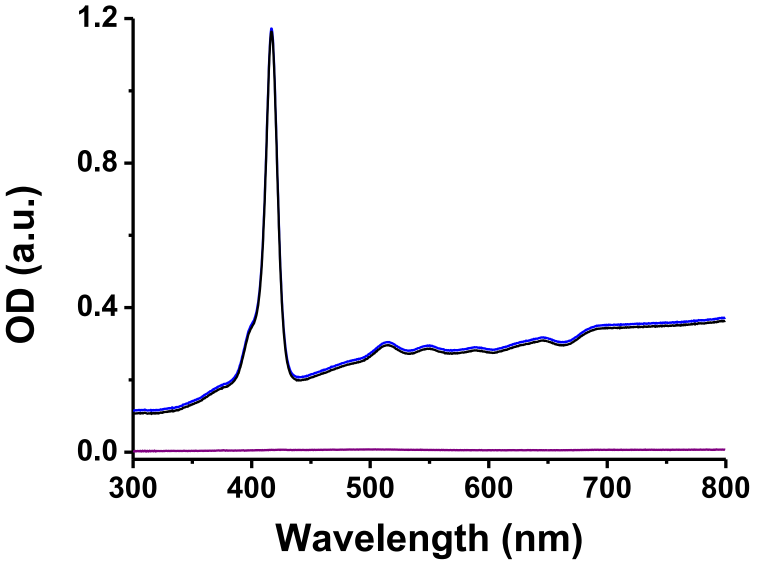

Figure S1. UV-Vis spectrum of the purified H_2P-MoS_2 hybrid material **2** (blue). Blue and purple lines represent the spectra acquired for the recovered solid (redispersed in DMF) and the supernatant after washing the H_2P-MoS_2 **2** with DMF under sonication, followed by centrifugation.

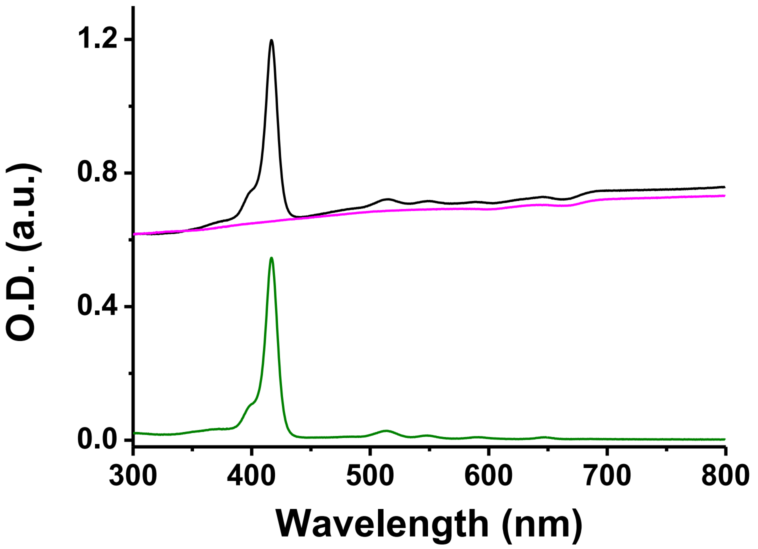

Figure S2. UV-Vis spectra of a mixture containing tetraphenylporphyrin (H_2TPP) and exfoliated MoS_2 (black). Magenta and olive lines represent the isolated solid (corresponds to the blank H_2TPP/MoS_2 physisorbed material) and the filtrate, respectively, after being processed likewise H_2P-MoS_2 **2**.

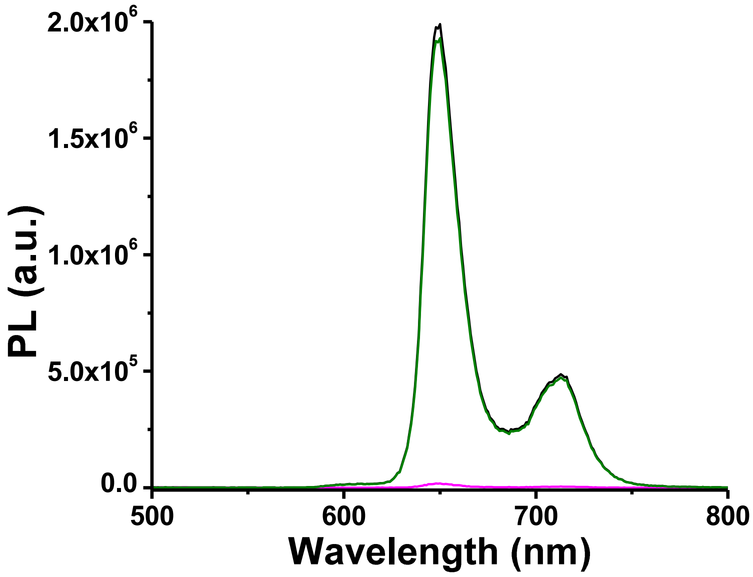

Figure S3. Photoluminescence spectra of a mixture containing tetraphenylporphyrin (H_2TPP) and exfoliated MoS_2 (black). Magenta and olive lines represent the isolated solid (corresponds to the blank H_2TPP/MoS_2 physisorbed material) and the filtrate, respectively, after being processed likewise H_2P-MoS_2 **2**.

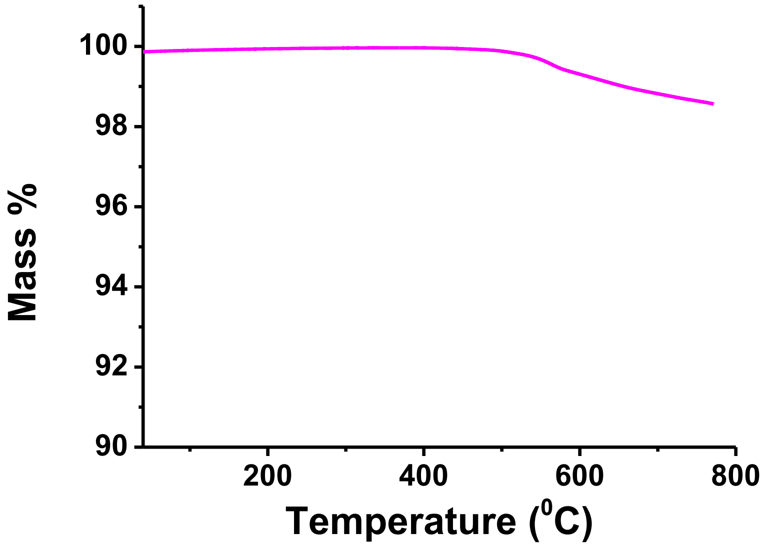

Figure S4. TGA graph of the isolated solid (corresponds to the blank H_2TPP/MoS_2 physisorbed material) derived by processing a mixture containing tetraphenylporphyrin (H_2TPP) and exfoliated MoS_2, after being processed likewise H_2P-MoS_2 **2**.

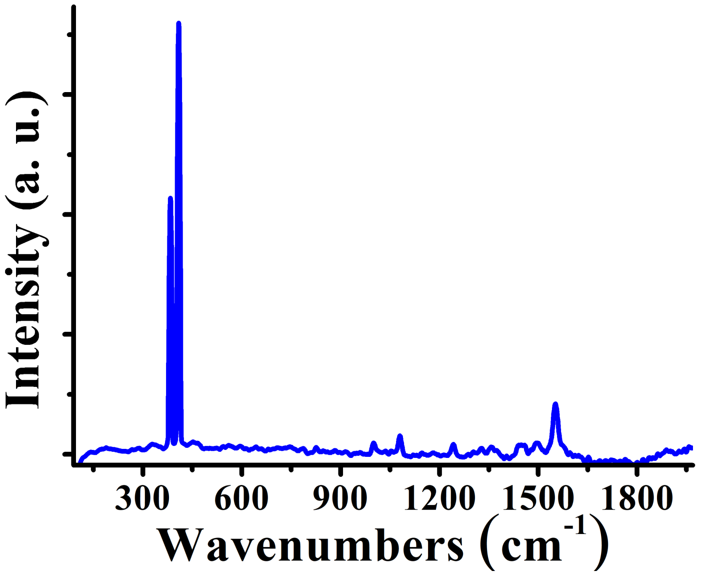

Figure S5. Raman spectrum (514 nm) of H_2P-MoS_2 hybrid material **2**.

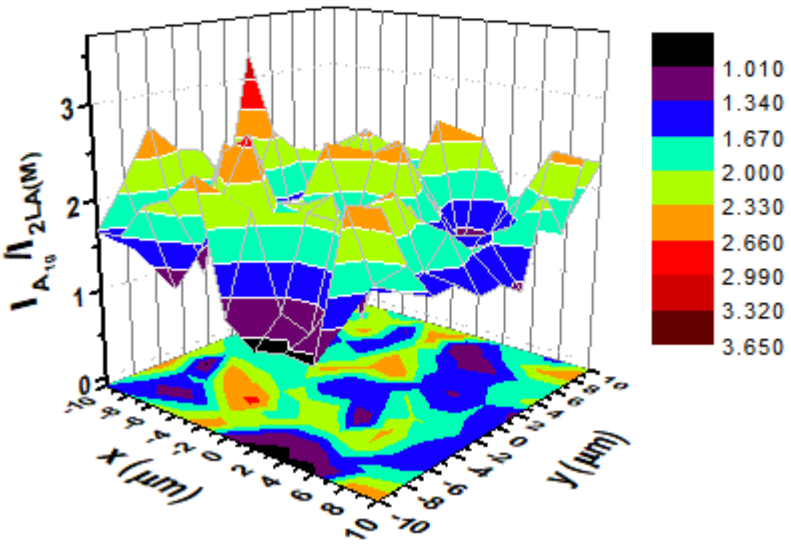

Figure S6. Raman mapping upon excitation at 633 nm of the $I_{A1g}/I_{2LA(M)}$ intensity ratio of a 20µm x 20µm area for the blank H_2TPP/MoS_2 physisorbed material

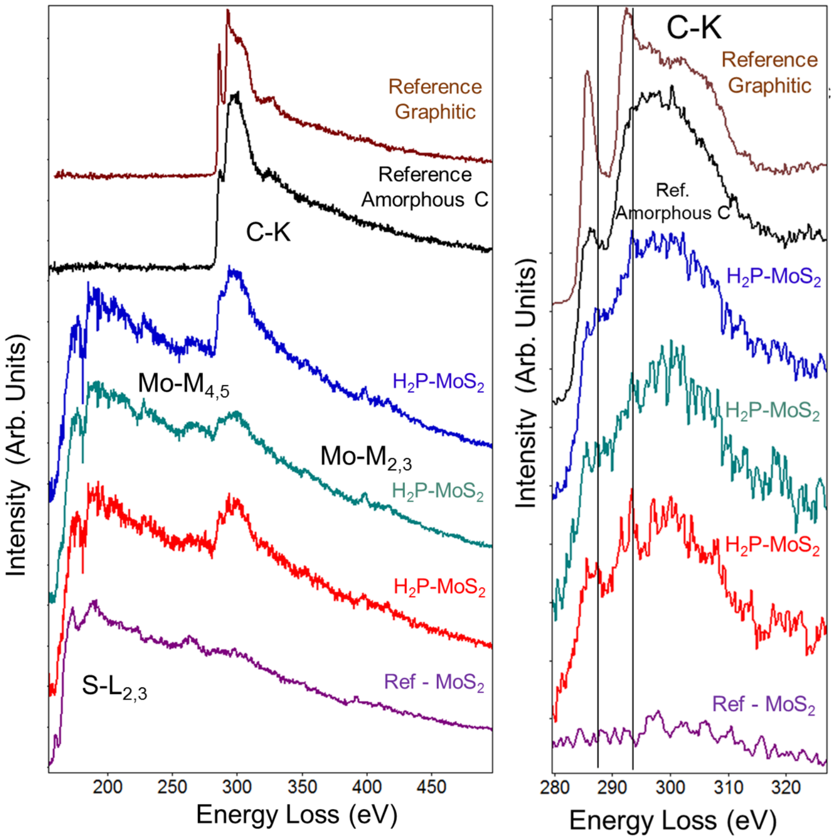

Figure S7. Left panel: selection of EEL spectra collected (from top to bottom) on a reference of pure sp^2 graphitic material, the amorphous carbon membrane of the TEM grid, three different areas of the H_2P-MoS_2 flakes, and a reference pristine MoS_2 flake. The S-L, Mo-M and C-K edges are observed in some of them depending the materials. Right panel: C-K edge, after background subtraction of the EELS spectra displayed on the left. Two different features have been highlighted via two lines at ~287.2 eV and ~293.4 eV, respectively.

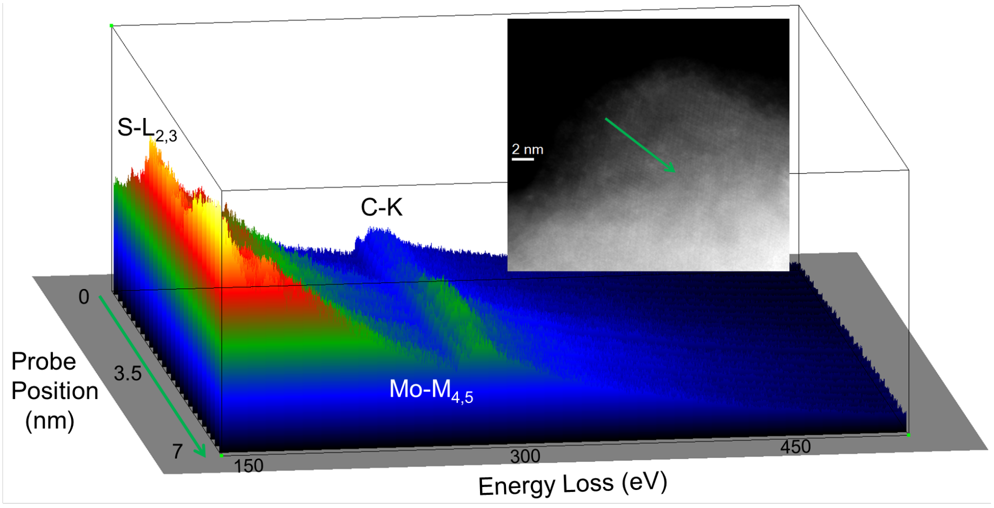

Figure S8. Spectrum-line (SPLI) EELS recorded on a H_2P-MoS_2 flake following the green line marked on the inset HAADF-STEM image. The arrow indicates the direction of the SPLI-EELS acquisition. S-L, Mo-M and C-K edges are observed. We can observe that these elements are uniformly distributed indicating an homogeneous repartition of the H_2P moieties at the surface of the edges of MoS_2 flakes.

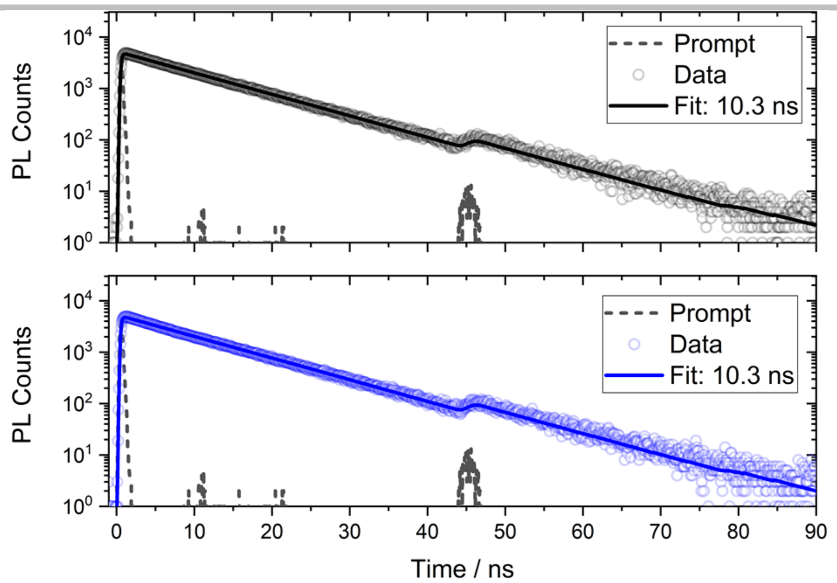

Figure S9. TCSPC emission lifetime measurements of **1** (top) and **2** (bottom) in DMF at room temperature upon 420 nm excitation.

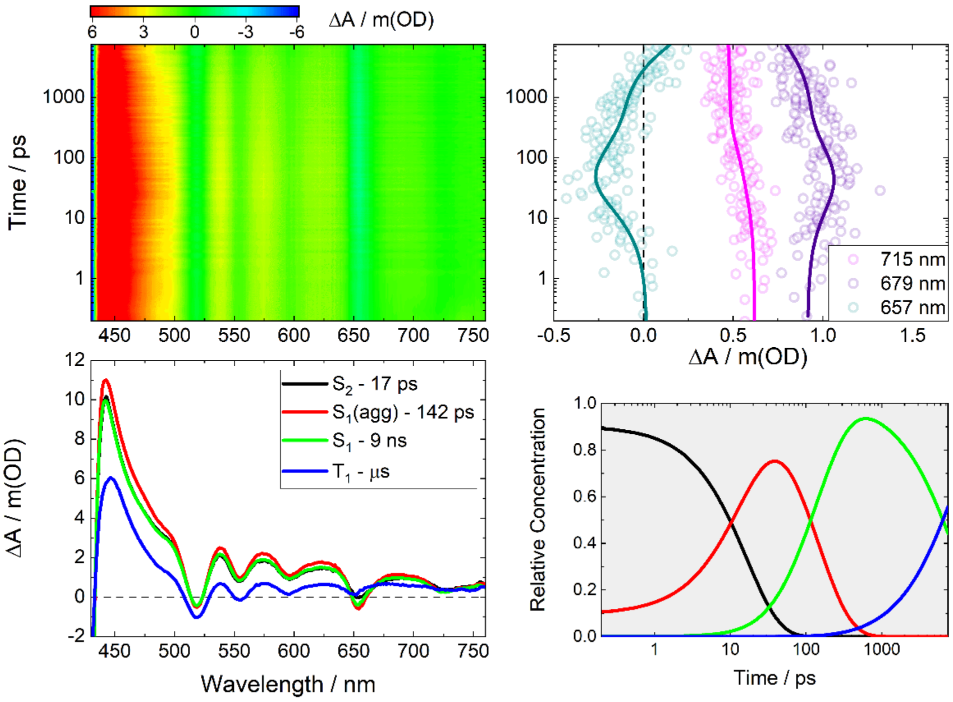

Figure S10. Upper left: Differential absorption 3D map obtained upon fsTAS of **1** in DMF at room temperature with 420 nm excitation. Upper right: Time absorption profiles and fits at selected wavelengths. Bottom left: Species associated differential spectra of S_2 (black curve), S_1(agg) (red curve), S_1 (green curve) and T_1 (blue curve). Bottom right: Concentration evolution of the different species over time.

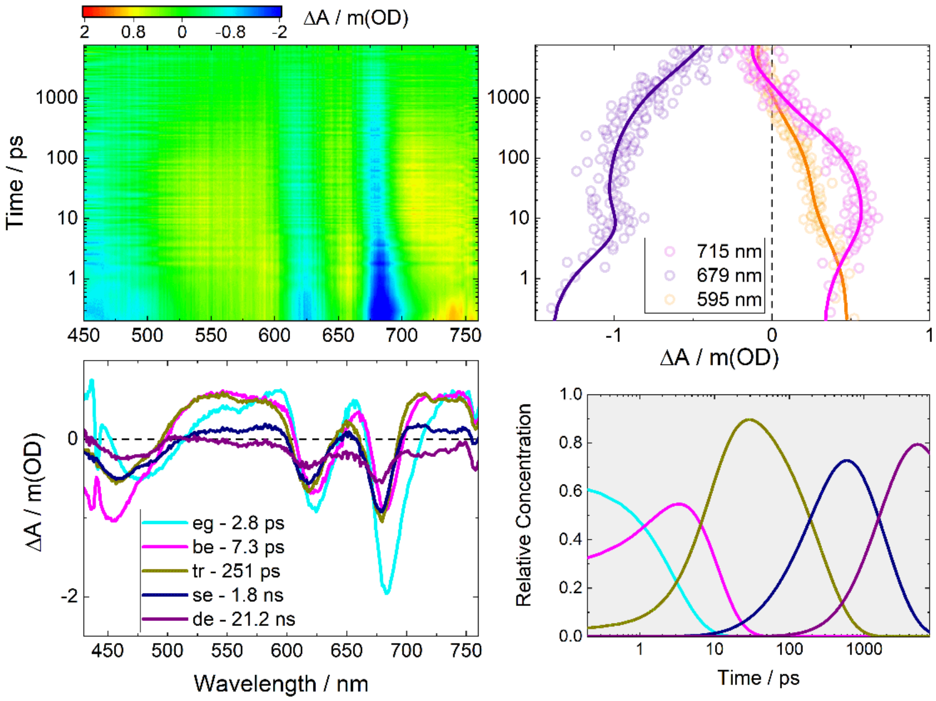

Figure S11. Upper left: Differential absorption 3D map obtained upon fsTAS of MoS_2 in DMF at room temperature with 420 nm excitation. Upper right: Time absorption profiles and fits at selected wavelengths. Bottom left: Species associated differential spectra of exciton generation (cyan curve), biexcitons (pink curve), trions (brown curve), single excitons (dark blue curve) and diffused excitons (purple curve). Bottom right: Concentration evolution of the different species over time.
